# Exclusion of Disperse Orange 3 is possible from the textile dye mix present in the Swedish baseline patch test series. A study by the Swedish Contact Dermatitis Research Group

**DOI:** 10.1111/cod.14223

**Published:** 2022-10-04

**Authors:** Marléne Isaksson, Annarita Antelmi, Jakob Dahlin, Joanna Stenton, Cecilia Svedman, Erik Zimerson, Bo Glas, Lina Hagvall, Maria Lagrelius, Anna Löwnertz, Laura Malinauskiene, Mihaly Matura, Magnus Bruze

**Affiliations:** ^1^ Department of Occupational and Environmental Dermatology Lund University, Skane University Hospital Malmö Sweden; ^2^ Department of Public Health and Clinical Medicine, Dermatology and Venereology Umeå University Umeå Sweden; ^3^ Department of Dermatology Sahlgrenska Academy at the University of Gothenburg Gothenburg Sweden; ^4^ Occupational and Environmental Dermatology, CAMM Stockholm County Council and Institute of Environmental Medicine, Karolinska Institute Stockholm Sweden; ^5^ Division of Dermatology and Venereology Ryhov County Hospital Jönköping Sweden; ^6^ Faculty of Medicine Institute of Clinical Medicine, Clinic of Chest Diseases, Immunology and Allergology, Vilnius University Vilnius Lithuania; ^7^ Department of Dermatology Skaraborgs Hospital Skövde Sweden

**Keywords:** allergic contact dermatitis, contact allergy, delayed hypersensitivity, disperse dyes, patch testing, textile dermatitis

## Abstract

**Background:**

The textile dye mix (TDM) 6.6% in petrolatum contains Disperse Blue (DB) 35, Disperse Yellow 3, Disperse Orange (DO) 1 and 3, Disperse Red 1 and 17, and DB 106 and 124. The most frequent allergen in TDM‐positive patients is DO 3. Around 85% of *para*‐phenylenediamine (PPD)‐allergic dermatitis patients have been positive to DO 3. There has been a discussion to exclude DO 3 from TDM 6.6% because of strong simultaneous reactions to TDM and PPD.

**Objectives:**

To study if DO 3 can be excluded from TDM 6.6%.

**Methods:**

Patch tests were performed on 1481 dermatitis patients with TDM 6.6%, TDM 7.0% (without DO 3 but the other disperse dyes at 1.0% each), DO 3 1.0%, and PPD 1.0% pet.

**Results:**

Contact allergy to TDM 6.6% was 3.6% and to TDM 7.0% was 3.0%. All 26 DO 3‐positive patients were positive to PPD. The 44 patients positive to TDM 7.0% plus the 13 positive to PPD and TDM 6.6% but negative to TDM 7.0% were 57, outnumbering the 53 positive to TDM 6.6%.

**Conclusion:**

TDM 7.0% can replace TDM 6.6% in the Swedish baseline series, since TDM 7.0% together with PPD 1.0% will detect patients with textile dye allergy.

## Graphical abstract

- PPD is a marker of Disperse Orange 3 (DO 3)‐allergy.
- Exclusion of DO 3 is possible from the textile dye mix (TDM 6.6% pet.) since those allergic to textile dye mix 7.0% (44) plus those allergic to PPD and TDM 6.6% but negative to TDM 7.0% (13) outnumbers those allergic to TDM 6.6% (53).

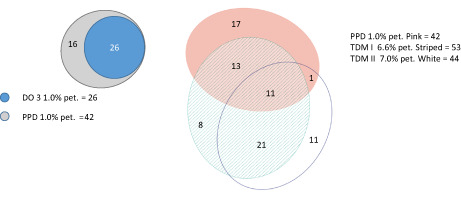

## 1 INTRODUCTION

Disperse dyes (DDs) are used for colouring synthetic textile fibres such as polyester, acrylic, acetate and polyamide. DDs are well‐known contact sensitizers and eight dyes are included in a textile dye mix (TDM) 6.6% in petrolatum (pet.) (in this article called TDM I) in the Swedish, European and International Contact Dermatitis Research Group baseline patch‐test series since 2015, 2016 and 2019, respectively.^1^, ^2^, ^3^, ^4^ In Table 1, the 8 dyes present in TDM I are presented with their respective concentrations in the mix. The share of positive reactions to this mix is around 2%–4%.^1^, ^5^

**TABLE 1:** The composition of textile dye mix (TDM) I and TDM II

Specific textile dye mixes	Name of dye and concentration in %	All in petrolatum
TDM I	Disperse Blue (DB) 35 DB 106 DB 124 Disperse Yellow (DY) 3 Disperse Orange (DO) 1 DO 3 Disperse Red (DR) 1 DR 17	1.0 0.3 0.3 1.0 1.0 1.0 1.0 1.0
TDM II	DB 35 DB 106 DB 124 DY 3 DO 1 DR 1 DR 17	1.0 1.0 1.0 1.0 1.0 1.0 1.0

Disperse Orange 3 (DO 3) was reported to be the most frequent dye allergen in TDM‐positive patients being patch tested with the ingredients of the mix.^1^, ^3^ Between 65% and 85% of *para*‐phenylenediamine (PPD)‐allergic patients have shown positive patch test reactions to DO 3^6^, ^7^, ^8^ and over 90% of patients positive to DO 3 react to PPD.^1^, ^9^ There has been a discussion to exclude DO 3 from TDM I because of frequent, strong and simultaneous patch‐test reactions to TDM I and PPD in allergic individuals.^1^, ^9^

A recent study at the department of Occupational and Environmental Dermatology in Malmö showed that 94.1% of the DO 3 – allergic dermatitis patients showed positive patch test reactions to PPD.^10^

However, in that study patch testing had not been simultaneously performed with DO 3 1.0% and PPD 1.0% in all patients. Our primary aim in the present study was therefore to patch test the two substances simultaneously together with TDM I and a TDM without DO 3 in consecutive dermatitis patients. The mix without DO 3 contained seven DDs at 1.0% each (altogether 7.0%), which in this article is named TDM II (Table 1). The purpose was to see if the mix without DO 3 together with PPD detected as many or possibly even more allergic patients compared to the original TDM I, if this mix was deleted from the baseline series. Another aim was to investigate the clinical relevance of the TDM allergy. The results will form the basis for deciding whether the present TDM in the Swedish baseline series should be replaced by a modified TDM without DO 3.

## 2 METHODS

### 2.1 Study population

The study was conducted by the Swedish Contact Dermatitis Research Group (SCDRG). Six Swedish dermatology clinics and additionally a Lithuanian collaborative clinic took part during the period 1 April 2020 till either 31 March 2021 or 30 June 2021. The intention was to test for a year. The participating clinics were located in Malmö, Jönköping/Nässjö/Värnamo, Stockholm, Umeå, Gothenburg and Skövde, all in Sweden, and in Vilnius, Lithuania. Results are based on the consecutive patch testing of 1481 dermatitis patients, 1055 females (mean age 42.3 years; range 7–87 years) and 426 males (mean age 43.7 years; range 5–91 years; female/male 71.2/28.8%). The local ethical committee of Lund had approved the study and participants signed a written informed consent (No. 2019‐04815).

### 2.2 Patch test preparations

Patch‐test preparations used in the study are listed in Table 2. All preparations were bought from Chemotechnique Diagnostics (Vellinge, Sweden) by the Malmö department and distributed to the participating clinics. All patch‐test preparations used in the study were made from the same batches, respectively.

**TABLE 2:** The petrolatum patch‐test preparations used and the prevalence of positive reactions in the study population

Patch‐test preparation	Contact allergy rate %	Contact allergy rate females (%)	Contact allergy rate males (%)	*p*‐value
TDM I (w/w)	3.6	41/1055 (3.9)	12/426 (2.8)	0.36
TDM II (w/w)	3.0	34/1055 (3.2)	10/426 (2.3)	0.40
PPD 1.0% (w/w)	2.8	35/1055 (3.3)	7/426 (1.6)	0.09
DO 3 1.0% (w/w)	1.8	22/1055 (2.1)	4/426 (0.9)	0.19

Abbreviations: DO, Disperse Orange; PPD, *para*‐phenylenediamine; TDM, textile dye mix; w/w/, weight/weight.

### 2.3 Patch testing

The patch testing and reading of the patients followed the routine of the participating clinics. Finn Chambers (8 mm diameter; SmartPractice, Phoenix, AZ, USA) on Scanpor tape (Norgesplaster, Vennesla, Norway) were used in all centres except Umeå, Gothenburg and Skövde, which used IQ Ultra chambers (8 × 8 mm; Chemotechnique Diagnostics) on a hypoallergenic surgical tape. The dose for the petrolatum preparations is 20 mg for a Finn Chamber (Ø8 mm)^11^ and 25 mg for the IQ Ultra chamber. The chambers were applied on the back and stayed occluded during 48 h. Readings were classified according to the ICDRG guidelines.^12^, ^13^ The definition of ‘positive’ is +, ++, +++ reactions only, excluding doubtful and irritant reactions.

All patients were read twice, on day (D)3 or D4 and on D7. For each patient with any patch test reaction (allergic, doubtful or irritant) an individual test protocol was filled in with data on initials, age, gender and patch test reactions to at least one of the following test preparations: TDM I, TDM II, PPD 1.0% and DO 3 1.0%. It was emphasized that all patch test reactions without an obvious morphology of an allergic or irritant nature must be classified as doubtful. Moreover, for each patient the following information was also to be noted: known present exposure to coloured synthetic textiles/garments or PPD and if known, source of information, and type of exposure (occupational or nonoccupational). An assessment of the present clinical relevance of the contact allergy to TDM or PPD were to be registered and the reading dermatologist had 1 of 4 options to tick, namely, explains the dermatitis (a dermatitis connected to either a positive test to TDM or PPD, respectively), aggravates/contributes to the dermatitis, has no influence on the dermatitis, or has unknown influence on the dermatitis. Late reactions beyond D8 were also to be reported. A dermatologist read all patch tests on both days in all centres in Sweden except Umeå, where a nurse trained in patch‐test readings did the first reading and a dermatologist the second one. In Vilnius, an allergologist trained in patch test techniques performed the readings. Any positive reaction (+, ++, +++) either on D3 or D4 or on D7 was registered as a positive reaction.

### 2.4 Statistics

McNemar's two‐sided test was used to compare the rates of contact allergy to TDM I and TDM II. Fisher's exact test, two‐sided, was used to compare the number of contact allergy cases in females and males and dichotomized positive test reactions. A *p*‐value of <0.05 was considered to be significant.

Three‐part Venn diagrams were calculated using EulerAPE.^14^

## 3 RESULTS

**FIGURE 1:**
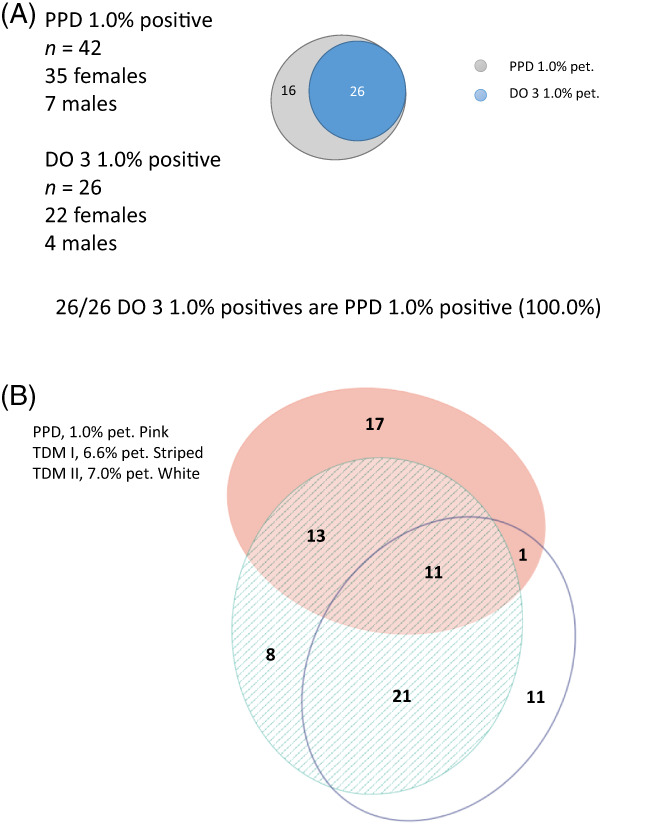
The distribution of exclusive and concurrent positive patch test reactions to *para*‐phenylenediamine (PPD) 1.0%, Disperse Orange 3 (DO 3) 1.0%, textile dye mix (TDM) I and TDM II on either day (D) 3/4 or D7 in 1481 dermatitis patients, presented as area proportional Euler diagrams. (A) Concomitant and solitary positive reactions to PPD 1.0% and DO 3 1.0%. (B) Concomitant and solitary positive reactions to PPD 1.0%, DO 3 1.0%, TDM I and TDM II. Forty‐four TDM II‐positive reactors plus those 13 reacting to PPD 1.0% and TDM I add up to 57 individuals that are detected as being textile dye allergic compared to 53 individuals detected by TDM I

Of the 1481 tested patients, 53 (3.6%) were positive to TDM I (41 females, 12 males) and 44 (3.0%) were positive to TDM II (34 females, 10 males) with no significant difference in females versus males (Tables 2 and 3; Table S1). Thirty‐two (2.2%) reacted simultaneously positive to TDM I and TDM II. Twelve patients were positive to TDM II but not to TDM I, and 21 were positive to TDM I but not to TDM II. Comparing the frequencies gave a *p* = 0.016. Thirteen patients reacted simultaneously positive to TDM I and PPD but negatively to TDM II. A total of 42 patients (2.8%) had a positive reaction to PPD with no significant difference in females versus males (Table 2). Twenty‐six (1.8%) reacted positively to DO 3 with no significant difference in females versus males (Table 2) and all 26 did also react to PPD (100%) (Figure 1A). Eight patients had a positive test reaction to TDM I but were negative to TDM II and PPD (Figure 1B).

**TABLE 3:** Number of tested patients and number of positive patients tested with textile dye mix (TDM) I and TDM II, contemporary reactors to PPD and their gender distribution

	Patients positive (*n*) to TDM I		Patients positive (*n*) to TDM II	
Total tested patients (*n* = 1481)	53, 24/53 are PPD 1.0% positive (45.3%)		44, 12/44 are PPD 1.0% positive (27.3%)	
Number of positive females and males	41 females	12 males	34 females	10 males

We did not detect any patients with a late reaction to any of the tested preparations that could be a sign of patch test sensitization.

In Tables S2a and b, the noted information in the individual protocols is summarized.

For synthetic textiles/garments exposure, in eight cases the contact allergy to either TDM I or TDM II was considered to explain or aggravate the dermatitis compared to 56 cases with contact allergy to either TDM I or TDM II and in which no influence or unknown influence on the dermatitis was considered. In Table 4A, the strength of the positive patch test reactions to the TDMs in those 8 and 56 patients, respectively, is seen. Results from both TDM I and TDM II are summarized and only the strongest patch test reaction is noted. If the sum of the 3‐plus and 2‐plus reactions taken together is compared to the sum of the 1‐plus reactions, the difference is not significant (*p* = 0.46) for TDM I and/or TDM II.

**TABLE 4:** Number and strength of positive (A) textile dye mix (TDM) and (B) *para‐*phenylenediamine (PPD) reactions in those patients considered to have a clinical relevance concerning their TDM reactivity and PPD allergy and those where the contact allergy was considered to have no influence on the dermatitis or have unknown influence on the dermatitis

(A)					
Eight patients in which the TDM allergy was considered to explain the dermatitis or aggravate/contribute to the dermatitis			56 patients in which the TDM allergy was considered to have no influence on the dermatitis or have unknown influence on the dermatitis		
TDM +++	TDM ++	TDM +	TDM +++	TDM ++	TDM +
3	1	4	14	22	20

(B)					
14 patients in which the PPD allergy was considered to explain the dermatitis or aggravate/contribute to the dermatitis			25 patients in which the PPD allergy was considered to have no influence on the dermatitis or have unknown influence on the dermatitis		
PPD +++	PPD ++	PPD +	PPD +++	PPD ++	PPD +
10	1	3	10	3	12

*Note*: Results from both TDM I and TDM II are summarized.

For PPD exposure, in 14 cases the contact allergy to PPD was considered to explain or aggravate the dermatitis compared to 25 cases with contact allergy to PPD in which no influence or unknown influence on the dermatitis was considered. In Table 4B, the strength of the positive patch test reactions to PPD in those 14 and 25 patients, respectively, is seen. If the sum of the 3‐plus and 2‐plus reactions taken together is compared to the sum of the 1‐plus reactions, the difference is not significant (*p* = 0.17) for PPD.

Four patients had had a black henna tattoo and in three a positive PPD‐reaction was seen. In Table S3, the PPD reactivity and clinical problems are shown.

## 4 DISCUSSION

The question of whether to remove DO 3 from TDM I has been discussed previously^1^, ^9^ and it has been suggested that it is DO 3 in the TDM I that causes the simultaneous and strong reactions to TDM and PPD, since these substances often give simultaneous reactions.^6^, ^7^, ^8^

Removing the DO 3 could lead to missed detection of DO 3 contact allergy. However, if a marker for DO 3 allergy was used for patch testing this could compensate by a later confirming test with DO 3. An obvious candidate for this marker function is PPD, why we tested DO 3 and PPD simultaneously in the same patients. Our results show that all DO 3 positive individuals also reacted to PPD, which would speak for exclusion of DO 3 from the TDM I (Figure 1A).

In a Swedish study from 2011, the same high number of concomitant reactions to PPD was seen, 27/28 = 96.4%.^1^ However, DO 3 was only tested when either TDM I or TDM 8.0% was positive. Another study within the European Environmental Contact Dermatitis Research Group showed similar results, 43/44 allergic to DO 3 also reacted to PPD (97.7%).^2^ The authors in both studies concluded that DO 3 may perhaps be excluded from the mix in the future but this would need further studies.^1^, ^2^ If DO 3 is excluded from TDM, and a patient reacts to PPD, which may indicate textile dye allergy, the patient should be questioned about skin problems from textiles and if these are/were present, DO 3 should be patch tested on its own, preferably included in a textile series. The patient information leaflet on PPD allergy must in that case be updated with accurate information.

Another question that arises is how the TDM without DO 3 should be composed. To elucidate this more carefully the following studies have been executed prior to this study.

In the aforementioned study from 2011,^1^ members of the Swedish Contact Dermatitis Research Group provisionally included a TDM at two concentrations, namely, 6.6% and 8.0% (all eight ingredients tested at 1.0%), in the baseline series of each centre, and dermatitis patients were tested with both simultaneously. Results showed no significant difference in the detection rate, although TDM 8.0% numerically detected more positive patients, and neither a higher frequency of irritant reactions nor other adverse reactions compared to testing with the mix 6.6% were seen.

Following this, an in‐clinic study was initiated in Malmö in 2017 to investigate whether a TDM without DO 3 could be used to avoid strong reactions to the TDM in patients with strong reactions to PPD. Two different TDMs without DO 3, one at 5.6% (Table 5) and the other one at 7.0% (Table 1) were tested in around 800 consecutively patch tested dermatitis patients during 1 year. We found that both mixes detected less positive patients than the original TDM I, which was also expected. The difference in detection rate between the two mixes without DO 3 was statistically not significant. TDM II detected numerically more positive reactors than TDM 5.6%. No active sensitization and virtually no irritant reactions were registered. We concluded that TDM II could be a suitable candidate for incorporation into baseline series to replace TDM I, but only if it was shown that virtually all patients with contact allergy to DO 3 were detected by PPD 1.0%, which our previous study had shown.^10^ Our aim in this study was therefore to patch test the textile dye mix TDM II.

**TABLE 5:** The composition of textile dye mix (TDM) 5.6%

TDM 5.6%	Name of dye and concentration in %	All in petrolatum
	Disperse Blue (DB) 35 DB 106 DB 124 Disperse Yellow (DY) 3 Disperse Orange (DO) 1 Disperse Red (DR) 1 DR 17	1.0 0.3 0.3 1.0 1.0 1.0 1.0

Actually, the present study shows that slightly more patients will be traced with the new TDM II provided that PPD‐allergic patients are considered as potential TDM/DO 3‐allergic patients. Fifty‐three individuals tested positively to TDM I. In these 53, 13 patients tested negatively to TDM II but positively to PPD. Thus, the sum of positive reactors to textile dyes based on a positive TDM II and a positive PPD in those 53 with a negative TDM II is 57 (44 + 13; Table S1) (Figure 1B). The present study shows that all DO 3 positive individuals reacted positively to PPD, which indicates that very few positive reactions will be missed by exclusion of DO 3 from the TDM (Figure 1A).

There has been an ongoing discussion on whether routinely patch testing with PPD 1.0% pet. is safe, owing to the risk of patch test sensitization.^15^ Late‐appearing patch test reactions may reflect patch test sensitization but may also be attributable to a low degree of pre‐existing sensitization.^15^

Hillen et al. reported a rate of 1.3% PPD reactions appearing after D7, which together with other PPD patch test data constituted the basis for the recommendation in Germany in 2006 to stop routine patch testing with PPD 1.0% pet., with a subsequent recommendation to lower the patch test concentration to 0.3% pet., to reduce the risk of active sensitization.^16^ Others have questioned these findings, stipulating that the risk of active sensitization has been over‐estimated, and have advocated continuing to use PPD 1% pet. For routine patch testing to avoid missing contact allergy to PPD.^17^, ^18^, ^19^, ^20^ A defined dose per unit area rather than concentration is crucial when the risk of patch test sensitization is evaluated. The elicitation and sensitization capacity depend not only on the concentration, but predominantly on the dose per unit area of allergen delivered to the skin and exposure time.^13^, ^21^ In a recent study, good agreement was shown for PPD patch test preparations with equivalent doses of 1.0% and 0.3% pet.^22^ If dosed correctly, PPD 1% pet. may be safe to patch test with.^15^

In several earlier screening studies, clinical relevance of contact allergy to TDM I has been found in around 20%–35% of positive patients.^1^, ^3^ In this study, however, of those 64 TDM‐positive individuals where clinical relevance was assessed and noted in the protocols, only 8/64 (12.5%) were considered to have a TDM allergy that explained or aggravated/contributed to the dermatitis (Table 4A). The reason for this low figure is not known. However, establishing a clinical relevance of contact allergy to textile dyes is generally considered to be difficult. There was also no significant difference in the strength of the positive test reactions +++ and ++ versus + in relation to clinical relevance, which could have been suspected if the contact allergy would have had clinical implications. Exposure to coloured synthetic textiles was to be noted, not exposure to the actual dyes present in the two TDMs, since this is impossible when this is not printed on the labels of garments. In the future maybe this will be the case. In a new study from Sweden 83 synthetic textiles from the Swedish market were analysed for the presence of DDs and the most common dye among the eight included in TDM was DO 3 (26%). According to results, other azo DDs than those included in TDM were generally more common.^23^ In a recent American study 21 azobenzene DDs were detected in children's polyester clothing purchased in the United States, 12 of which were confirmed and quantified via reference standards. Azo dyes were found to be present in apparel at total levels up to 11 430 μg dye/g shirt and at individual levels up to 9230 μg dye/g shirt (Disperse Red 354). However, not any of the 8 DDs present in TDM were detected.^24^

Concerning PPD allergy and clinical relevance in the 39 patients, (Table 4B) there was no significant difference in the strength of the positive test reactions +++ and ++ versus + in relation to clinical relevance, which could have been suspected if the contact allergy would have had clinical implications. However, in those four patients with a previous black henna tattoo, three were positive to PPD, and those with a strong PPD allergy, that is a +++ reaction, had experienced problems with hair dye, which is a known problem. The one with a + reaction to PPD had not experienced any problems from black henna hair dye containing PPD, which shows that weak contact allergies do not necessarily give skin problems (Table S3).

## 5 CONCLUSION

In this study, TDM II plus PPD detected slightly more positive patients than the original TDM I, 57/1481 versus 53/1481 (Table 3; Table S1). It is therefore proposed that TDM I be excluded from the Swedish baseline series and that TDM II is inserted instead. Based on this study, evaluation to do the same in the European baseline series could be done. Since PPD‐positivity may indicate textile dye allergy, skin problems from textiles must be evaluated and if present, DO 3 should be patch tested on its own, preferably included in a textile series. The patient information leaflet on PPD allergy must also be updated with accurate information. PPD 1.0% is already present in most baseline series, also the Swedish and the European, so there is no need to change that.

## AUTHOR CONTRIBUTIONS

All authors have participated sufficiently to take public responsibility for the work.

## CONFLICT OF INTEREST

The authors declare no conflicts of interest.

## Supporting information


**Table S1** Number of patients tested in each participating clinic and number of reactors to the tested substances.
**Table S2a**. Estimated exposure to disperse dyes in the 65 individuals testing positive to textile dye mix I and/or textile dye mix II.
**Table S2b**. Estimated exposure to *para‐*phenylenediamine (PPD) in the 42 individuals testing positive to PPD 1.0%.
**Table S3**. Skin problems related to black henna tattoos and hair dying and *para‐*phenylenediamine (PPD)‐reactivity in 3 patients with anamnesis of having had black henna tattoos.

## Data Availability

Research data are not shared.
