# Supplementary material for: Exclusion of Disperse Orange 3 is possible from the textile dye mix present in the Swedish baseline patch test series. A study by the Swedish Contact Dermatitis Research Group
Source: Contact Dermatitis. 2022 Oct 4;88(1):54–9. doi: 10.1111/cod.14223 (PMC10091765; doi:10.1111/cod.14223)
Supplement: Supplementary file 1 — Table S1 Number of patients tested in each participating clinic and number of reactors to the tested substances. Table S2a. Estimated exposure to disperse dyes in the 65 individuals testing positive to textile dye mix I and/or textile dye mix II. Table S2b. Estimated exposure to para‐phenylenediamine (PPD) in the 42 individuals testing positive to PPD 1.0%. Table S3. Skin problems related to black henna tattoos and hair dying and para‐phenylenediamine (PPD)‐reactivity in 3 patients with anamnesis of having had black henna tattoos. [file COD-88-54-s001.docx]

Supplement Table 1. Number of patients tested in each participating clinic and number of reactors to the tested substances.

| Total no of tested  Females/males | Positive to TDM I | Positive to TDM II | Positive to both TDM I and TDM II | Positive to TDM I  Positive to PPD 1.0%  Negative to TDM II | Positive to TDM II  Negative to TDM I | Positive to PPD 1.0% | Positive to DO 3 1.0% | Positive to both PPD 1.0% and  DO 3 1.0% |
| --- | --- | --- | --- | --- | --- | --- | --- | --- |
| Malmö  392  266/126 | 9 | 8 | 7 | 1 | 1 | 8 | 3 | 3 |
| Vilnius 194  171/23 | 11 | 7 | 4 | 4 | 3 | 6 | 6 | 6 |
| Umeå  182  132/50 | 7 | 3 | 3 | 1 | 0 | 3 | 1 | 1 |
| Stockholm 70  46/24 | 6 | 5 | 3 | 2 | 2 | 8 | 4 | 4 |
| Gothenburg 333  215/118 | 10 | 6 | 5 | 5 | 1 | 12 | 9 | 9 |
| Jönköping  160  108/52 | 3 | 6 | 3 | 0 | 3 | 1 | 0 | 0 |
| Skövde  150  117/33 | 7 | 9 | 7 | 0 | 2 | 4 | 3 | 3 |
| Total  1481  1055/426 | 53 | 44 | 32 | 13 | 12 | 42 | 26 | 26 |

TDM I= Textile dye mix 6.6%; TDM II= Textile dye mix 7.0%; PPD= *para*-phenylenediamine; DO 3= Disperse Orange 3.

Supplement Table 2a. Estimated exposure to disperse dyes in the 65 individuals testing positive to textile dye mix I and/or textile dye mix II.

| Known present exposure to colored synthetic textiles/garments in 63/65 individuals | | | | If known exposure, source of information-textile dye mix | | Type of exposure (product) textile dye mix | | Relationship between present exposure and dermatitis in 64/65 individuals – textile dye mix | | | |
| --- | --- | --- | --- | --- | --- | --- | --- | --- | --- | --- | --- |
| Yes | Possibly | No | Unknown | Anamnesis | Dermatologist´s own experience | Occupational | Non-occupational | Explains the dermatitis | Aggravates/contributes to the dermatitis | No influence on the dermatitis | Unknown influence on the dermatitis |
| 23 | 12 | 4 | 24 | 13 | 16 | 3 | 48 (1 had both) | 2 | 6 | 20 | 36 |

Supplement Table 2b. Estimated exposure to *para-*phenylenediamine (PPD) in the 42 individuals testing positive to PPD 1.0%.

| Known present exposure to PPD in 41/42 individuals | | | | If known exposure, source of information- *para-*phenylenediamine | | Type of exposure (product) - *para-*phenylenediamine | | Relationship between present exposure and dermatitis in 39/42 individuals - *para-*phenylenediamine | | | |
| --- | --- | --- | --- | --- | --- | --- | --- | --- | --- | --- | --- |
| Yes | Possibly | No | Unknown | Anamnesis | Dermatologist´s own experience | Occupational | Non-occupational | Explains the dermatitis | Aggravates/contributes to the dermatitis | No influence on the dermatitis | Unknown influence on the dermatitis |
| 18 | 3 | 12 | 8 | 18 | 8 | 3 | 29 | 11 | 3 | 12 | 13 |

Supplement Table 3. Skin problems related to black henna tattoos and hair dying and *para-*phenylenediamine (PPD)-reactivity in 3 patients with anamnesis of having had black henna tattoos.

| Patient no. | Eventual skin problems connected to the tattoo earlier or at the time of the study | Eventual hair dye problems earlier or at the time of the study | PPD^×^ reactivity |
| --- | --- | --- | --- |
| 1 | No skin problems earlier or at the time of the study | No problems from black henna hair dye at the time of the study | + |
| 2 | Had had a rash from the black henna tattoo earlier | Had had a rash from hair dye, used no dye at the time of the study | +++ |
| 3 | No skin problems earlier or at the time of the study | Problems from hair dye at the time of the study | +++ |

^×=^ *para-*phenylenediamine
